# New Perspectives on Antiacne Plant Drugs: Contribution to Modern Therapeutics

**DOI:** 10.1155/2014/301304

**Published:** 2014-07-24

**Authors:** Priyam Sinha, Shruti Srivastava, Nidhi Mishra, Narayan Prasad Yadav

**Affiliations:** Herbal Medicinal Products Department, CSIR-Central Institute of Medicinal and Aromatic Plants (CSIR-CIMAP), P.O. CIMAP, Lucknow 226015, India

## Abstract

Acne is a common but serious skin disease, which affects approximately 80% adolescents and young adults in 11–30 age group. 42.5% of men and 50.9% of women continue to suffer from this disease into their twenties. Bacterial resistance is now at the alarming stage due to the irrational use of antibiotics. Hence, search for new lead molecule/bioactive and rational delivery of the existing drug (for better therapeutic effect) to the site of action is the need of the hour. Plants and plant-derived products have been an integral part of health care system since time immemorial. Therefore, plants that are currently used for the treatment of acne and those with a high potential are summarized in the present review. Most active plant extracts, namely, *P. granatum, M. alba, A. anomala,* and *M. aquifolium* exhibit minimum inhibitory concentration (MIC) in the range of 4–50 *µ*g/mL against *P. acnes,* while aromatic oils of *C. obovoides, C. natsudaidai, C. japonica,* and *C. nardus* possess MICs 0.005–0.6 *μ*L/mL and phytomolecules such as rhodomyrtone, pulsaquinone, hydropulsaquinone, honokiol, magnolol, xanthohumol lupulones, chebulagic acid and rhinacanthin-C show MIC in the range of 0.5–12.5 *μ*g/mL. Novel drug delivery strategies of important plant leads in the treatment of acne have also been discussed.

## 1. Introduction

### 1.1. General

The comorbidity of chronic skin conditions and mental health disorders has long been known among the emerging groups of psychodermatology and neurodermatology. Recurrently, acne vulgaris is a common dermatological condition allied with depression, anxiety, and other psychological sequelae [1]. Acne is one of the most common multifactorial chronic inflammatory diseases of the pilosebaceous follicles involving androgen induced sebaceous hyperplasia, altered follicular keratinisation, hormonal imbalance, immune hypersensitivity, and bacterial (*Propionibacterium acnes*) colonisation [2, 3]. Although acne lacks the urgency of a life-threatening condition without impairing the overall fitness, it produces long term ramifications that can be momentous coming up with cutaneous and emotional scars lasting lifetime [4]. It hampers an individual's confidence causing physical, social, and psychological sufferings and reduces self-esteem and emotional distress caused by perceived disfigurement [5, 6].

The clinical manifestations of acne include seborrhoea (excess grease), noninflammatory lesions (open and closed comedones), inflammatory lesions (papules and pustules), and various degrees of scarring due to cyst formation [2]. The distribution of acne corresponds to the highest density of pilosebaceous units; it is distributed over face, neck, upper chest, shoulders, and back. According to the lesion type, acne can be classified into noninflammatory (purely comedonal acne) and inflammatory acne (mild papular, scarring papular, and nodular). Grading upon its severity, it can be categorized into mild, moderate, and severe acne. Mild acne comprises of open and closed comedones (<20), inflammatory lesions (<15) with total lesions not exceeding 30. Likewise, in moderate acne numerous papules and pustules are observed along with comedones (20–100), inflammatory lesions (15–50) whereas total lesions in the range of 30–125. Severe acne is diagnosed with extensive lesions including nodules and scarring together with cysts (>5), total comedone count (>100), total inflammatory count (>50) and total number of lesions more than 125 [7, 8].

### 1.2. Epidemilogy

Virtually, no mortality is evidenced in this disease, but there is often noteworthy physical and psychological morbidity. On the word of statistics, globally around 85% of young adults aged 12–25 years old, approximately 8% of adults aged 25–34 years old, and 3% of adults aged 35–44 years old experience certain degree of acne [9]. On an average 42.5% of men and 50.9% of women continue to suffer from the disease in their twenties [5]. Recent findings concluded that, in 30% of women, acne can persist during their entire fertile period [10]. Affecting 40 to 50 million people in USA per year, a significant number of adults continue to struggle with acne even after their teenage years. One population study in Germany found that 64% of individuals 20 to 29 years old and 43% of individuals 30 to 39 years old had visible acne. Another study from Germany of more than 2000 adults showed that 3% of men and 5% of women still had definite mild acne at the age of 40 to 49 years [11]. In a study of 309 subjects in southern India, the closed comedones differed from open comedones by a factor of 4.9 : 1. A total of 186 patients (60.2%) had grade 1 acne vulgaris and 85 (27.5%), 8 (2.6%), and 30 (9.7%) patients had grades 2, 3, and 4, respectively, [12]. Recently, it was noted that heritability of acne is almost 80% in first degree relatives and is more severe in those with a positive family history. Acne was found to be more frequent and severe among smokers following a dose dependent association [13]. The burden of acne in terms of cost to society was not well demarcated, but its predominance endorsed the high costs bestowing a substantial financial burden to the community. In a recent report in USA, the cost of acne is estimated to be 3 billion dollars per year in terms of treatment and loss of productivity [11].

### 1.3. Pathophysiology of Acne

The multifactorial pathogenesis of acne instigates at the pilosebaceous unit that consisted of multilobulated sebaceous glands, an epithelial lined follicular canal, and a hair [14]. Pathophysiology of acne is attributed to different notable factors such as sebaceous gland hyperplasia with seborrhoea, alteration in the quality of sebum lipids, inflammatory processes besides immune response, dysregulation of the hormone microenvironment, interaction with neuropeptides, and follicular hyperkeratinization followed by proliferation of* Propionibacterium acnes* within the follicle [15, 16].

The relation between androgen level and sebum production in acne vulgaris has been preestablished. The first factor in the genesis of acne is the androgen induced hypertrophy of sebaceous glands with overproduction of sebum [17]. The sebaceous glands possess steroid metabolizing enzymes which convert dehydroepiandrosterone (DHEAS) to dihydrotestosterone (DHT). Furthermore, two subtypes of 5-*α*-reductase isozymes, that is, type 1 and type 2, expressed in the scalp, chest, sebaceous glands, genitourinary tissue, and dermal papillae as well as in hair follicles, convert testosterone to the more active DHT [5]. Excess sebum production causes occlusion in the pilosebaceous unit and increases cell turnover in the follicular canal. Moreover, in the second factor of pathogenesis, pilosebaceous follicles are surrounded by macrophages and inflammatory mediators expressing Toll like receptors (TLR2) on their surface. TLR2 activation leads to transcription of nuclear factor triggering and thus heading towards the expression of cytokines, such as interleukin-1*β* (IL-1*β*), IL-8, and granulocyte macrophage-colony stimulating factor (GM-CSF), initiates and propagates the inflammatory response that further induces keratinocyte hyperproliferation [18]. Retention of desquamated keratinocytes within the pilosebaceous unit initiates follicular plugging and obstruction which triggers obliteration of the normal architecture of the follicle and formation of a thin-walled cystic lesion that is the comedo. As the keratinocytes and sebum continue to accumulate, the microcomedo wall eventually ruptures prompting inflammation [19].

Developing comedone covers greasy plugs comprising mixture of keratin, sebum, bacteria, and the superficial layer of melanin which may appear as a black head or a white head. Comedones when outburst through skin surface having central black appearance (due to the oxidation of tyrosine to melanin by tyrosinase) are called “black heads” or open comedones. However, impaction and distension of the follicle with improperly desquamated keratinocytes and sebum result in the development of “white heads” or closed comedones which remain underneath the skin surface as closed follicles [3, 20]. Depending upon the severity of pathologic conditions, these lesions represent a papule, pustule, nodule, and cyst.

*Propionibacterium acnes* is an anaerobic Gram-positive bacterium that produces propionic and acetic acid. In the follicular infundibulum of comedones, a large number of* P. acnes *are observed because comedones are filled with a lipid substrate as a nutrient source proposing it as an ideal site for anaerobes. Ultrastructural observation shows that* P. acnes *are 0.4 to 0.7 *μ*m in width and 3 to 5 *μ*m in length, possessing a ribosome rich cytoplasm and a relatively thick cell wall composed of peptidoglycan [17].* P. acnes* is involved in the development of inflammatory acne by activating complements and metabolizing sebaceous triglycerides into fatty acids that irritate the follicular wall and surrounding dermis. It also produces exoenzymes and chemotactically attracts neutrophils [4].* P. acnes* produces lipases, proteases, and hydrolases, contributing to inflammation and tissue destruction; expresses stress proteins which are responsible for comedonal rupture; and also incites an inflammatory response by acting on TLR-2. This may stimulate the expression of cytokines, such as IL-6 and IL-8 by follicular keratinocytes and IL-8 and IL-12 in macrophages, which is thought to activate hyperkeratinization, cell adhesion, follicular obstruction, and inflammation. The sequential phenomena lead to vascular and cellular events of inflammatory response and cause follicular disruption giving rise to acneiform lesions in the form of papules, pustules, and nodules.

### 1.4. Molecular Targets for Acne Treatment

Human sebocytes are biologically and metabolically very active cells and consequently express numerous receptors [21]. Many ligands bind to these receptors and produce varied responses altering the cell proliferation, cytokine production, and lipid synthesis, ultimately involved directly or indirectly in acne pathogenesis. After reviewing the literature, possible ligands have been enlisted, which trigger cell proliferation, cytokine expression, and lipogenesis after binding with their respective receptors. Individual ligand (agonist) has its own mode of action causing acne and hence requires specific antagonist which will bind to these receptors and cure acne. As far as acne treatment and management are concerned, all these pathways should be considered and recognised prior to treatment selection.

(1)* Neuropeptides* like vasoactive intestinal polypeptide (VPAC), neuropeptide Y and calcitonin gene-related peptide, and Substance P usually bind on VPAC receptors which are present on sebocytes of sebaceous glands. The consequences of binding are cytokines expression, sebocyte proliferation/differentiation, and upregulation of lipogenesis [16, 17, 21].

(2)* PRAR ligands* are such as leukotriene B4 (5-lipoxygenation product derived from arachidonic acid), a well-known natural ligand of PPAR*α* receptors present in mitochondria, peroxisomes, and microsomes of sebocytes. This ligand is known to induce lipogenesis in cultured human sebocytes and thereby, 5-lipoxygenase inhibitors could be considered to reduce lipogenesis and acne lesions.

(3)* Histamine *bound to the histamine 1 receptor and induces squalene synthesis in SZ95 sebocytes. As a result, lipid peroxidation takes place and squalene peroxide is produced as a byproduct. Further, it induces inflammatory responses as well ascomedogenesis and sometime interferes with sebocytesdifferentiation and sebogenesis [21, 22].

(4)* Insulin in higher concentration and insulin like growth factors-1 *activate IGF-I receptor which is expressed on SZ95 sebocyte cell surface. IGF-I amplifies lipid accumulation in sebocytes in a dose dependent manner. It is also known to stimulate5*α*-reductase, adrenal and gonadal androgen synthesis, androgenreceptor signal transduction, and hence sebocyte proliferation [23, 24].

(5)* Fibroblast growth factor* (FGF) secreted by keratinocyte derived interleukin-1*α* stimulated fibroblast and binds on FGF receptor 2b present in suprabasal spinous layer of the epidermis and sebocytes. FGF plays a crucial role in controlling epithelial proliferation and differentiation. At the same time androgen mediated upregulation of FGFR2b signalling is also possible which brings out follicular hyperkeratinization and sebaceous gland hypertrophy thereby [25, 26].

(6)* Corticotrophin releasing hormone* and urocortin bind to the CRH-receptor 1 (CRH-R1) at human sebocytes and reduces sebocyte proliferation, upregulates 3*β*-hydroxysteroid dehydrogenase, stimulates lipogenesis and keratinocyte differentiation, and increases in local inflammation by expressing IL-6 and IL-8 [27–29].

(7) *β* *-Endorphin* binds with *μ* opiate receptors present on sebaceous glands which stimulates lipogenesis and specifically increases the amount of fatty acids to an extent similar to linoleic acid in sebocytes.

(8) *α* *-Melanocyte stimulating hormone* binds at melanocortin 1 and 5 receptors (MC-1R and MC-5R), located at the cellular surface of sebocytes. MC-1R regulates inflammation in SZ95 sebocytes and exhibits a stronger expression in acne involved sebaceous glands.

(9)* Retinoic acid (RA) and 9-cis retinoic acid *are the ligands of retinoic acid receptors (RAR*α* and *γ*) and retinoid X receptors (RXR*α*) which are the predominant retinoid receptors in human sebocytes that regulate cell proliferation/differentiation.

(10)* Vitamin D*_3_ binds to vitamin D receptors and induces time and dose dependent modulation of cell proliferation, cell cycle regulation, lipid content, and IL6 and IL8 secretion by cultured sebocytes.

(11)* P. acnes* moieties stimulate TLR receptors (TLR-2,4,6) on keratinocytes. TLR activation results in the release of inflammatory cytokines (TNF-*α*, IL-6,and IL-8) by keratinocytes [16, 21].

(12)* Matrix metalloproteinases *are present in sebum and originate in keratinocytes and sebocytes. Sebum contains MMP-1, MMP-13, TIMP-1, TIMP-2, proMMP-9, and MMP-13, among which the latter two decreased with isotretinoin parallel to clinical improvement. The drug inhibited the arachidonic acid, induced secretion and mRNA expression of MMPs in HaCaT keratinocytes [30, 31].

(13)* Dipeptidyl peptidase IV and aminopeptidase N* are ectopeptidases which bind on particular receptors and participatein regulation of sebocytes. DP IV and APN enhance proliferation, reduce terminal differentiation, and stimulate total neutral lipid production. Furthermore, these promoted proliferation and IL-2 production of* P. acnes* stimulated T cells* ex vivo* and suppressed the expression of the immunosuppressive cytokine transforming growth factor-*β*1 [32, 33].

Altered lipogenesis, sebum production, hyperkeratinization, proliferation/differentiation of sebocytes, and cytokine expression may be reason for the formation of acne lesions. Acne management could be done efficiently only when the exact mechanism involved in the pathogenesis is known. The above targets could be considered during the assessment of the antiacne potential of the eminent active moieties in near future because these mechanisms play direct or indirect role in the acne pathogenesis.

## 2. Challenges in Acne Treatment

The management of acne is a long-standing process which must be customized to each patient. After the diagnosis of the disease, a suitable therapeutic strategy is the root of its treatment. Based on the type and severity of acne, the selection of proper medication depending on its mechanism of action relating its ability to address one or more of the pathogenic factors is the major challenge allied with its treatment selection. In this context, treatment of acne renders several challenges inspite of numerous therapeutic agents available.

### 2.1. Antibiotic Resistance

The persistent relevance of antibiotics in acne treatment is coupled with the risk of emerging resistant bacteria. The increase in antibiotic resistance is multifactorial, involving the specific nature of the relationship of bacteria to antibiotics. Consequently, there are adequate motives to search for alternative remedies to solve this problem. To overcome antibiotic resistance as well as the high treatment cost, medicinal plants have been studied as alternative treatments for acne.

### 2.2. To Surmount the Glitches Allied with Conventional Formulations of Antiacne Drugs

The followup phase of management requires a framework for approaching treatment modification that may include concepts such as nonexistence of effectual system for delivery of antiacne drugs. Antiacne drugs incorporated in conventional system either cannot reach the pilosebaceous unit at defined concentration or may not release the active moiety leading to their subtherapeutic levels. The problem can be resolved by employing futuristic approaches that is targeting the active molecule directly to the pilosebaceous unit or sebaceous gland which can eradicate the underlying microbial flora of* P. acnes* and inflammatory mediators responsible for acne vulgaris. Novel drug delivery system (NDDS) may be a preference to minimize the glitches related to conventional formulations like variation in drug efficacy and absorption, physicochemical characteristics of the active molecules and carriers or their improper incorporation in the conventional vehicles.

### 2.3. Non-Availability of Appropriate Animal Model

A major limitation in the development of an appropriate drug and delivery system for acne is the unavailability of a suitable animal model that can certainly mimic various pathophysiologic characteristics in humans.

## 3. Current Management Approaches

Grounded on the type and severity of acne lesions, rational use of existing treatment choices is currently an essential component of successful acne therapy. Mainstream acne management associates topical treatment either as monotherapy or in combination with a systemic drug therapy depending on the severity of acne. The current armamentarium available consists mainly of topical and oral retinoids, topical antimicrobials, systemic antibiotics, keratolytics, and hormonal therapy that consisted of oral contraceptives as well as androgen blocking agents, in addition to combination therapy of all the aforementioned agents.

Topical retinoids (vitamin A derivatives) are comedolytic agents which reduce abnormal mitosis of keratinocytes, hyperkeratinization, and inflammation. Modified slow release formulations and a third generation retinoid adapalene are reported to be less irritating. Azelaic acid is a naturally occurring dicarboxylic acid with modest antibacterial and comedolytic effects. Erythromycin and clindamycin are the most commonly used topical antibiotics in acne. They are useful in inflamed lesions with associated antibiotic resistant as a major problem. Oral antibiotics, namely, tetracyclines and macrolides, are prescribed in moderate to severe inflammatory acne, thereby precluding the practicality of applying topical therapies. In addition to the above mentioned antibiotics, trimethoprim, sulfamethoxazole, and ciprofloxacin are also used. In multiple trials, topical combination therapies are more effective than monotherapy as they are capable of targeting multiple pathogenic mechanisms. A number of fixed dose topical combination products are available including adapalene-BPO (0.1%/2.5%), clindamycin-BPO (1%/5% gel), erythromycin-BPO (3%/5% gel), erythromycin-tretinoin (4%/0.025% solution), and clindamycin-tretinoin (1.2%/0.025% gel). Oral contraceptives, including ethinyl estradiol in combination with cyproterone acetate, levonorgestrel, norgestimate, desogestrel, drospirenone, and ethynodiol diacetate, inhibit serum androgen levels, increase sex hormone binding globulin, and improve acne regardless of the type of progestin or concentration of estrogen. A detailed overview of these therapeutic approaches together with their mode of action and associated adverse effects [34–42] has been tabulated in Table 1.

## 4. Plants Having Antiacne Potential

The expedition for measures to combat acne continues to be a major research and development initiative in pharmaceutical and personal care industries [43]. The sustained application of antibiotics entails the risk of emerging resistant bacteria which is needless to mention. The development of antibiotic resistance is multifactorial, involving the specific nature of the relationship of bacteria to antibiotics [44]. Subsequently, there are sufficient purposes for searching alternative remedies that work out and resolve these problems. To overcome antibiotic resistance as well as the high treatment cost, medicinal plants have been studied as alternative treatments for diseases. As an alternative approach, numerous reports have indicated the possibility of using medicinally potent plant actives to counter the growth of the bacteria and inflammatory response. Occurrence of 250,000–500,000 plant species offers a great potential for screening phytotherapeutic agents which can be utilized for acne management. Traditional herbal medicines provide an interesting and largely unexplored source for the development of new drugs. Traditional medicines and natural products offer a great hope in the identification of bioactive lead compounds and their development into drugs for the treatment of acne vulgaris [20]. An attempt is also being made to enumerate the possible leads from traditional medicinal system for the treatment of acne with an ongoing search for novel biologically active botanical agents.

### 4.1. Plant Extracts

Plant extracts are therapeutically desired; medicinally active portions of medicinal plants are separated from inactive or inert components using selective solvents by standard extraction procedures like decoction, maceration, infusion, digestion, percolation, and soxhlet extraction. These are obtained in the form of decoctions, infusions, tinctures, semisolid, and powdered extracts. Some of the active plant extracts with antiacne properties have been discussed below.

*Echinacea purpurea *extract provided antiacne effect by inhibiting proliferation of* P. acnes* and reversing the bacterial induced inflammation. It also normalized elevated cytokine levels including IL-6 and IL-8 (CXCL8) in cell culture models of human bronchial epithelial cells and skin fibroblasts through cytokine antibody arrays [45].* Garcinia mangostana* is well known for its marked antiacne potential, and the dichloromethane extract of the pericarp exhibited the most potent antibacterial effect against both* P. acnes *and* S. epidermidis* with highest amount of *α*-mangosteen as quantified by HPLC. The MIC values for* P. acnes* and* S. epidermidis* were the same (0.039 mg/mL), whereas the MBC values were 0.039 and 0.156 mg/mL, respectively, [46]. The extracts reduced the TNF-*α* production as determined by ELISA, effective in scavenging free radicals, and suppressed the production of proinflammatory cytokines [47]. In addition to these plants,* Senna alata* (0.625–2.5 mg/mL MIC),* Eupatorium odoratum *(0.625 mg/mL MIC), and* Barleria lupulina *(1.25–2.5 mg/mL MIC) also showed strong inhibitory effects against* P. acnes* based on broth dilution method [44].

Moreover,* Camellia sinensis *polysaccharide showed strong inhibitory activities against hemagglutination mediated by pathogens* H. pylori*,* P. acnes, *and* S. aureus *with MIC in the range of 0.01–0.5 mg/mL. Findings proposed that the adhesion of these pathogens to hostcell lines exhibited selective antiadhesive effect of* C. sinensis* only against* P. acnes* pathogens [48]. Furthermore, 3% of green tea extract emulsion was recognised to reduce skin sebum production in healthy human volunteers using sebumeter [49]. In a comparative study, methanolic extract of* C. sinensis* possessed utmost antibacterial activity against* S. aureus*,* S. epidermis,* and* P. acnes* (MIC 1.25 mg/mL), when compared to extracts of* Glycyrrhiza glabra* and* Calendula officinalis* by agar disc diffusion method (due to the presence of alkaloids, flavonoids, glycosides, and terpenoids) recommending them as responsible phytoconstituents for the antiacne activity [50].

Additionally,* Punica granatum* rind extract containing 13% ellagic acid exhibited potent bacteriostatic effect against* P. acnes* (MIC 15.6 *μ*g/mL),* S. aureus,* and* S. epidermidis* (MIC 7.8–15.6 *μ*g/mL). It also inhibits nitric oxide production by murine macrophage like RAW 264.7 cells and the release of b-hexosaminidase from antigen stimulated rat basophilic leukemia cells revealing its antiallergic properties [51]. Potent extract of* Psidium guajava *and* Juglans regia *leaf extracts showed* in vitro *inhibitory effect on* P. acnes* and other organisms isolated from acne lesions of thirty-eight patients by disk diffusion method [52].* Selaginella involvens *extract inhibited nitric oxide production, iNOS/IL-1*β* expression, and cytokines (IL-1*α* and IL-8) in keratinocytes along with antioxidant effect in a dose dependent manner [53].* Terminalia arjuna *bark extract with flavonoid and tannin fractions were tested against* P. acnes *and* S. epidermidis*, in which flavonoid fraction (MIC 0.315 mg/mL) and its 2% cream formulation were found to be more effective [54].

The extracts and formulations of white tea, witch hazel, and rose have a protective effect on fibroblast cells and elicited a significant decrease in the amount of IL-8 produced by fibroblast cells against hydrogen peroxide induced damage. In addition to this, white tea and rose also showed considerable anticollagenase, antielastase, and antioxidant activities [55]. Methanolic extracts of* Rosa damascene*,* Eucommia ulmoides,* and* Ilex paraguariensis* were found to inhibit the growth of* P. acnes* with respective MICs of 2, 0.5, and 1 mg/mL. In addition, the latter two reduced the secretion of proinflammatory cytokines such as tumour TNF-*α*, IL-8, and IL-1*β* by human monocytic THP-1 cells pretreated with heat killed* P. acnes* at a concentration of 0.1 mg/mL [56]. In a study,* Rubia cordifolia, Curcuma longa, Hemidesmus indicus,* and* Azadirachta indica* extracts caused significant suppression of reactive oxygen species from polymorphonuclear leukocytes and proinflammatory cytokine induced monocytes [57].* Coscinium fenestratum *extract with MIC values of 0.049 mg/mL against* P. acnes* and* S. epidermidis* and MBC values of 0.049 and 0.165 mg/mL, respectively, proved its antiacne activity. Similarly,* Tephrosia purpurea, Euphorbia hirta, Curcubito pepo, *and* Eclipta alba *had strong inhibitory effects against* P. acnes* [58]. Leaves and seeds of* Borago officinalis*,* Linum bienne,* and* Ruta graveolens* and aerial parts of* Malva sylvestris* and* Rubus ulmifolius* were known to effectively treat acne [59, 60].

*Morus alba* root extract showed MIC values 15.6 *μ*g/mL and 3.1 *μ*g/mL against* P. acnes* and* S. epidermidis,* respectively. Extracts from* Phellodendron amurense*,* Albizzia julibrissin*, and* Poncirus trifoliate* also produced remarkably low MIC values against both the pathogens [61]. Likewise,* Anacardium pulsatilla *containing polyphenols and* Podocarpus nagi* containing flavonols are recognized to be effective against* P. acnes* [62]. Another well-known plant is* Angelica anomala* which had strong inhibitory effects against* P. acnes* and* S. epidermidis* with MIC values of 15.6 *μ*g/mL and 126 *μ*g/mL, respectively.* Mollugo pentaphylla*,* Matteuccia orientalis,* and* Orixa japonica* also inhibited the growth of both pathogens, along with the reduction in* P. acnes* induced secretion of IL-8 and TNF-*α* in THP-1 cells [63].* Caesalpinia sappan *and* Intsia palembanica* were noted as potent antiacne plants based on their antibacterial (MIC 0.13 mg/mL; MBC 0.25 mg/mL), lipase inhibitory, and antioxidative properties [64].

The organic extracts of* Elephantorrhiza elephantina*,* Ekebergia capensis, Eucalyptus camaldulensis,* and* Harpephyllum caffrum* displayed noteworthy activity against* P. acnes* with MIC values between 0.05 and 1.00 mg/mL [65]. In another study, ethanolic extracts of* Ammnia baccifera*,* Hibiscus syriacus*,* Quercus infectoria*,* Berberis aristata*,* Couroupita guianensis,* and* Symplocos racemosa* have shown antiacne potential, but* Symplocos racemosa *was found to be most effective with MIC value of 0.044 mg/mL [66].* Pisum sativum *seeds containing proteins, lecithins, carbohydrates, and* Trifolium pretense *having isoflavones are used in treating acne [67].

*Vitex agnus-castus* has been shown to be effective in the treatment of premenstrual acne. Antioxidative and antimicrobial properties of* Usnea barbata*,* Solanum dulcamara, *and* Saccharomyces cerevisiae* are responsible for their antiacne potential [68]. A methanol-dichloromethane extract of different* Eucalyptus *species, namely,* E. globulus*,* E. maculata*, and* E. viminalis *possessed potent antiacne activity. Oregon grape crude root extracts and its alkaloids berberine and jatrorrhizine showed MIC values between 5 and 50 *μ*g/mL against* P. acnes in vitro* [69]. A gel formulation containing 0.1% of anthraquinone rich fraction from the roots of* R. cordifolia* exhibited potent activity against* P. acnes*,* S. epidermidis,* and* M. furfur* when compared with standard clindamycin gel by cup plate diffusion method [70]. In another study, MIC value of the extract against* P. acnes* was found to be 600 *μ*g/mL by broth dilution method and produced a significant zone of inhibition [71].

Terpenoids of* Gossypium barbadens,* possess antiacne properties owing to its antimicrobial and antioxidant effects.* Eucommia ulmoides* was found to inhibit* P. acnes* with MIC 0.5 mg/mL and reduce the secretion of proinflammatory cytokines. Radical scavenging activity of* Phyllanthus emblica*, COX-2, and NO expression inhibiting activity of* Aralia continentalis*, inhibitory effect on lipid peroxidation by* Clerodendrum indicum, * and suppression of PGE2 production by* Clerodendron trichotomum* enables their use against inflammatory acne [72, 73]. Additionally, the extracts obtained from* Aglaia roxburghiana *fruits,* Euonymus pendulus *bark,* Emblica officinalis *fruits, and* Raphanus sativus* were also active and inhibited lipid peroxidation [73, 74].* Lygodium japonicum* extracted by the pressure assisted water extraction noticeably possessed antioxidant and antibacterial activity with MIC 2.59, 3.33, and 7.37 mg/mL against* Listeria monocytogenes*,* Salmonella typhimurium,* and* P. acnes*, respectively, [75].

### 4.2. Essential Oils

Natural essential oil, a concentrated hydrophobic liquid containing volatile aroma compounds, is obtained from plant organs by water distillation, steam distillation, and cohobation in addition to enfleurage method. Recently, these essential oils have also been explored for their wide application in acne management.

A single-blind, randomized clinical trial on 124 patients with mild to moderate acne concluded that both 5%* Melaleuca alternifolia* (tea tree oil) and 5% benzoyl peroxide lotion encouragingly reduced the number of inflamed and noninflamed lesions with considerably fewer side effects from tea tree oil [76]. In tea tree oil, terpinen-4-o1, *α*-terpineol, and *α*-pinene were found to be active against* S. aureus*,* S. epidermidis,* and* P. acnes* [77]. From a study of 60 patients with mild to moderate acne, tea tree oil in terms of total acne lesion count and acne severity index was found to be highly effectual in treating acne [78]. Similarly, the essential oil of* Zingiber cassumunar* (Plai oil) exhibits antimicrobial activity against a wide range of bacteria (MBC 0.62% against* P. acnes*), dermatophytes, and yeasts, confirming its potential application against acne [79].

Moreover,* Thymus quinquecostatus *essential oil retains considerable antibacterial, antioxidant, antielastase, and anti-inflammatory effects against acne inducing bacteria. It also induces low cytotoxicity in human cell lines proving itspossible effectiveness for acne treatment [80]. Additionally, antibacterial activity of rosemary essential oil against* P. acnes* (MIC 0.56 mg/mL) was probably attributed to its bioactive components such as 1,8-cineole, *α*-pinene, camphor, and camphene. The atomic force microscopy and phase images confirmed that, at lower concentration, rosemary essential oil attached to the surface of bacterial cell and with increase in concentration, the bacterial bodies were severely damaged [81].

Furthermore, volatile oils from* Eucalyptus globulus* (MIC and MBC 9.38 mg/mL) and* Psidium guajava* leaves (MIC 9.38 mg/mL, MBC 37.50 mg/mL) exhibited antimicrobial activity as determined by agar diffusion and microdilution methods against* P. acnes *due to *γ*-terpinene and *α*-pinene [82]. The* in vivo *rat sebaceous gland model concluded that eucalyptus oil decreases sebum production by reducing the size of sebaceous glands, thus controlling the spread of acne [83].

Essential oil of* Abies koreana* possessed excellent antibacterial activities against drug susceptible and resistant* P. acnes* and* S. epidermidis*. In addition, it reduced the LPS-induced secretion of TNF-*α*, IL-1*β*, IL-6, NO, and PGE2 in RAW 264.7 cells, indicating its anti-inflammatory effects [84]. Similarly, the antibacterial activity of coriander oil against* P. acnes *and* S. epidermidis *was investigated and MIC values were found to be 1% and 1.1% v/v, respectively, by agar dilution method [85].* In vitro* antiacne potential of* Citrus obovoides* and* Citrus natsudaidai *oils was proved as they exhibited antibacterial activity against* P. acnes* and* S. epidermis* (MIC 0.31 *μ*L/mL, & 2.5–10 *μ*L/mL) as well as superoxide anion radical scavenging activity. They also reduced* P. acnes* induced secretion of IL-8 and TNF-*α* in THP-1 cells [86].* Cryptomeria japonica *essential oil, containing kaurene, enemol, *γ*-eudesmol, and sabinene, has potent antimicrobial activity against* P. acnes* and* S. epidermidis* (MIC 0.16–10 *μ*L/mL) and has inhibitory effects on NO, PGE_2_, TNF*α*, IL-1*β*, and IL-6 production in lipopolysaccharide activated macrophages [87].

Another well-known* Ocimum gratissimum *oil preparation in a cetomacrogol blend base was well tolerated, was more effective, and reduced acne lesions faster than benzoyl peroxide 10% lotion showing its effectiveness in the management of acne [88]. It was indicated that aloe vera gel enhanced the antiacne properties of Ocimum oil, and their combination is more effective than 1% clindamycin in the treatment of acne [89]. From a study, it was concluded that essential oils of* Cymbopogon nardus*,* Cymbopogon citratus*,* Citrus hystrix*,* Ocimum sanctum*,* Ocimum basilicum, Zingiber cassumunar,* and* Zingiber officinale* possessed antimicrobial, anti-inflammatory, and antioxidant properties. Among these,* Cymbopogon nardus* oil (citronella oil) was most active against* P. acnes* (MIC 0.005–0.3 *μ*L/mL). In addition to this, all the essential oils except kaffir lime oil demonstrated notable free radical scavenging activity. The major components such as eugenol in holy basil oil and d-limonene in kaffir lime oil were suggested to contribute to this activity [90]. In another study, ten essential oils,* Mentha spicata*,* Zingiber officinale*,* Citrus limon*,* Citrus paradisi*,* Jasminum grandiflora*,* Lavandula angustifolia*,* Matricaria chamomilla*,* Thymus vulgaris*,* Rosa damascene, *and* Cinnamomum zeylanicum *were identified to have antiacne properties against* P. acnes*. It was witnessed that thyme, cinnamon, and rose essential oils exhibited the best antibacterial activities with MIC of 0.016%, 0.016%, and 0.031% v/v, respectively, [91].

In a study of ocimum oils,* Ocimum basilicum *(sweet basil) and* Ocimum sanctum* (holy basil) essential oils along with their microemulsions were screened for their* in vitro* activity against* P. acnes* using disc diffusion method indicating that the MIC values of sweet basil and holy basil oils were 2.0% and 3.0% v/v, whereas microemulsion of sweet basil oil possessed higher activity than that of holy basil oil [92]. Australian essential oil,* Backhousia citriodora,* was shown to possess significant antimicrobial activity against* S. aureus*,* E. coli*,* P. aeruginosa*,* C. albicans*, methicillin resistant* S. aureus*,* A. niger*,* K. pneumoniae,* and* P. acnes* [93].

Essential oils of* Citrus aurantium*,* Eucalyptus radiate*,* Juniperus communis*,* Pelargonium asperum*,* Pogostemon cablin,* and* Styrax benzoe* have been used in the treatment of acne [94]. In addition, essential oils of* Anthemis aciphylla*,* Salvia desoleana*, and* S. sclarea* showed weak to moderate inhibitory effect against* S. aureus* and* S. epidermidis*.* Tamarix bovena* essential oil was found to inhibit facial flora that would be applicable in acne treatment [95].

*Helianthus annuus* and* Cucurbita pepo* seed oils as well as flax or linseed oil, having mainly linoleic and linolenic acids, were used for dermatological treatments including acne. Besides the above mentioned natural oils,* Prunus armeniaca*,* Argania spinosa*,* Persea gratissima*,* Adansonia digitata*,* Ribes nigrum*,* Vaccinium macrocarpon*,* Zea mays*,* Oenothera biennis*,* Vitis vinifera*,* Corylus Americana*,* Schinziophyton rautanenii*,* Moringa oleifera*,* Elaeis guineensis*,* Papaver orientale*,* Brassica napus*,* Rubus idaeus*,* Oryza sativa*,* Carthamus tinctorius*,* Sesamum indicum*,* Glycine soja*,* Prunus amygdalus,* and* Juglans regia* oils are known to be used in the treatment of acne [72]. These scientific findings recommend the vital role of essential oils in possible customized therapeutic regimens for acne management.

### 4.3. Phytomolecules

Plants contain a broad range of medicinally active chemical components or phytoconstituents that account for their medicinal properties. The first target after obtaining an active extract is identification, isolation, and characterization of plant extract bioactive phytomolecule(s). Recently many scientific reports signify the applications of isolated phytomolecules in targeting acne lesions through various mechanisms which are summarized below.

Rhodomyrtone, principle compound of* Rhodomyrtus tomentosa* leaves, was tested against* P. acnes* (MIC 0.5 *μ*g/mL) using broth macrodilution method and was reported to be significantly effective by reducing 99% of the bacterial cells within 24 hours. Cytotoxicity test performed on human normal fibroblast indicated very low cytotoxicity favouring its use as topical therapeutic antiacne agent [96]. Pulsaquinone and hydropulsaquinone isolated from extract of the roots of* Pulsatilla koreana* exhibited antimicrobial activity against* P. acnes* with MIC values of 2.0 and 4.0 *μ*g/mL, respectively, [97]. Furthermore, Brazilin, protosappanin A, and sappanone B, isolated from methanolic extracts of* Caesalpinia sappan *wood, showed significant lipase inhibitory and antibacterial activity with MIC values of 0.50 mg/mL, 1.00 mg/mL, and >2.00 mg/mL, respectively. The antioxidant activity of brazilin and protosappanin A was also found to be appreciably higher than sappanone B [98]. Similarly, the ethyl acetate extract of* Momordica charantia* (wild bitter melons) contains phytol and lutein as bioactive constituents which strongly suppressed proinflammatory cytokine and MMP-9 levels in* P. acnes* stimulated THP-1 cells, attenuated ear swelling and granulomatous inflammation, and activated PRAR a and b in the transactivation assay [99]. Further* ent*-kaurene diterpenoids, namely, rosthornins A–D, isolated from the dried leaves ether extract of* Rabdosia rosthornii,* exhibited antibacterial activity specifically against* P. acnes* (3.17–25 *μ*g/mL) [100].

The active compound berberine from* Rhizoma coptidis *exerted anti-inflammatory effects through a negative regulation of COX-2 and API as well as by inhibition of lipoxygenases [101]. Its extract showed its anti-inflammatory effect by inhibiting the expression of various proinflammatory cytokines and cell surface molecules involved in inflammatory responses at the transcriptional level [102]. Flavonoids like kaempferol and quercetin, isolated from* Impatiens balsamina* in combination with clindamycin and erythromycin, were found to be potent against antibiotic resistant* P. acnes* with MIC values of ≤32 *μ*g/mL and ≤64 *μ*g/mL, respectively, [103]. Likewise, honokiol and magnolol isolated from* Magnolia spp*. evidenced potent antibacterial activities against* P. acnes* and* P. granulosum*, by disk diffusion method with MIC 3-4 *μ*g/mL and 9 *μ*g/mL, respectively. Additionally, their killing curve analysis showed that* P. acnes* was rapidly killed within 10 min of treatment at the rate of 105 organisms per mL. They also reduced the secretion of IL-8 and TNF-*α* induced by* P. acnes* in THP-1 cells indicating their anti-inflammatory effects [104].

A new abietane diterpene, 2b-acetoxyferruginol, isolated from the stem bark of* Prumnopitys andina, *had antibacterial activity assayed against* P. acnes* and was found to be most active at the concentration of 4 *μ*g/mL [105]. Panduratin A, a natural chalcone compound isolated from* Kaempferia pandurata, *has noteworthy* in vitro* antistaphylococcal activity against clinical staphylococcal isolates [106].* Mahonia aquifolium *stem bark crude extract and its protoberberine alkaloids, berberine, and jatrorrhizine revealed antimicrobial activity against twenty strains of coagulase negative staphylococci and* P. acnes* (MIC 5–50 *μ*g/mL) isolated from skin lesions of acne patients [107].* Epimedium brevicornum* and* Polygonum cuspidatum* extracts and their active compounds icariin, resveratrol, and salidroside were identified to possess marked antibiofilm activity against* P. acnes* when used in subinhibitory concentrations [108].

Naturally derived components, xanthohumol and lupulones from* Hupulus lumulus,* illustrated strong inhibitory activity against* P. acnes* (MIC 0.1–3 *μ*g/mL),* S. epidermidis*,* S. aureus*,* K. rhizophila,* and* S. pyogenes*. These compounds in addition to humulones demonstrated moderate to strong anticollagenase inhibitory activities. Xanthohumo, also showed the highest total oxygen radical absorbance capacity and singlet oxygen absorbance capacity thereby proving its antioxidative potential [109]. Flavonoids 2′,6′-dihydroxy-3′-methyl-4′-methoxy-dihydrochalcone, eucalyptin, and 8-desmethyl-eucalyptin, isolated from* E. maculata* extracts, considerably inhibited the growth of* S. aureus*, MRSA,* B. cereus*,* E. faecalis*,* A. acidoterrestris*,* P. acnes,* and* T. mentagrophytes*, with MIC ranging from 1.0 to 31 mg/L [110]. Several saponins and flavonoids, namely, *β*-aescin, digitonin, kaempferol, and catechin showed strong lipase inhibitory activity with low toxicity thereby confirming there use against acne [111].

Bakuchiol obtained from the edible seeds of* Psoralea corylifolia* showed strong antibacterial effects, anticollagenase, COX-2, COX-1, and expression of inducible nitric oxide synthase genes inhibitory activity. It has broad spectrum antioxidant activity, effectively quenches superoxide, hydroxy, peroxy, peroxynitrile radicals, and singlet oxygen non radicals in addition to inhibiting lipid peroxidation. A pilot clinical study showed that 1% bakuchiol reduced acne by a score of about 57%, whereas 2% salicylic acid only reduced acne by about 48%, but when used in combination it reduced acne lesions and inflammation upto 70% [112]. It has been reported that the fruits and root of* A. dahurica *containing imperatorin, phellopterin, xantoroxin, byakangelcol, oxypeucedanin, neobyakangelcol, and coumarin markedly suppressed neutrophil chemotaxis. Similarly,* Coptis chinensis* root and stem, containing high concentrations of berberine, have strong antilipogenic effect; furthermore,* G. glabra *containing glycyrrhizine, triterpene glycoside, glabric acid, flavanones, and isoflavones showed remarkable antibacterial activity against* P. acnes* [113].

Wogonin (5,7-dihydroxy-8-methoxyflavone) isolated from the methanolic extract of* Scutellaria baicalensis* potently lowered mRNA levels of COX-2, TNF-*α*, and PGE2 in a subchronic skin inflammation model of tetradecanoylphorbol-13-acetate induced ear edema. It also affected intercellular adhesion molecule-1 and IL-1b but to a lesser extent [114]. Rutaecarpine, a quinazolinocarboline alkaloid, evodiamine, dehydroevodiamine, and triterpenoid evodin, isolated from unripe fruit extract of* Evodia rutaecarpa,* inhibited ultraviolet A induced ROS generation, resulting in the enhanced expression of MMP-2 and MMP-9 in human skin cells. It also inhibited H_2_O_2_ induced increase in the expression of MMP-2 and MMP-9, COX-2, and phospholipase A2 [115]. Matrine, baicalin, ursolic acid, sodium danshensu, hesperidin, and andrographolide significantly reduced IL-8 and TNF-*α* by human HaCaT keratinocyte cells pretreated with heat-killed* P. acnes* [116]. Extracts from* Terminalia chebula* and* Embelia ribes* showed lipase inhibition, chebulagic acid being the responsible component for antilipase activity, with MIC of 12.5 *μ*g/mL against* P. acnes* [117]. Among various compounds isolated from the methanolic extract of the seeds of* Arctium lappa*, namely,isolappaol C, lappaol C, lappaol D, lappaol F, and diarctigenin, the latter two strongly inhibited NO production in the LPS-stimulated RAW264.7 cells [118].

Geraniin, isolated from* Phyllanthus embelica*, showed strong activity in DPPH and lipid peroxidation assays as well as NO scavenging activity [72, 119].* Anthemis nobilis *and* Matricaria recutita* were employed in skin inflammation due to their biologically active flavonoids, particularly apigenin, *α*-bisabolol, and chamazulene [72]. Rhinacanthin-C (82.59%) isolated from* Rhinacanthus nasutus* extract was quoted to have potent bactericidal activity against* S. mutans* and* P. acnes* with MIC values of 2–8 *μ*g/mL, respectively, [120].

A recent study explored the inhibitory potential of rosmarinic acid, a phenolic compound from* Rosmarinus officinalis*, with MIC values 62.5 *μ*g/mL and 31.25 *μ*g/mL against* P. acnes* and* S. aureus*, respectively, [121]. In a study, out of 10 flavonoids isolated from* Intsia palembanica* methanolic extract, fustin, ampelopsin and 4′-dehydroxyrobidanol were found to be the most active compounds to inhibit the lipase activity of* P. acnes* using the 2,3-dimercapto-1-propanol tributyrate (BALB) method with IC50 values of 13.7 *μ*M, 36.1 *μ*M, and 40.0 *μ*M, respectively, [122]. Epigallocatechin-3-gallate, the major polyphenol in green tea, was found to reduce sebum by modulating the AMPK-SREBP-1 signaling pathway and inflammation through suppression of NF-*κ*B and AP-1 pathways. These findings were supported by an 8-week randomized, split-face, and clinical trial where the molecule significantly improved acne and was found to be well tolerated [123].

Signs of acne are evident as dark spots (hyperpigmentation) and scars on the affected skin surface, which is due to overexpression and accumulation of melanin regulated by the enzyme tyrosinase. It was confirmed in a study that* Artocarpus integer *root extract possessed tyrosinase inhibition potential (90.57%) and antimicrobial activity against* S. aureus*,* S. epidermidis*,* P. acnes,* and* T. mentagophytes*. Compounds isolated from the above plant extract, artocarpin and cudraflavone C, showed the potent antibacterial activity against* S. aureus*,* S. epidermidis, *and* P. acnes* with MIC at 2, 4, and 2 *μ*g/mL, respectively, whereas artocarpanone exhibited antityrosinase potential [124].

The above discussion regarding the scope of natural therapeutics in the form of plant extracts, essential oils, and various isolated phytomolecules spell out the worth of plant derived treatment options against acne vulgaris. These findings also propose their broad relevance against* P. acnes*, directly or indirectly via similar mechanisms as that of synthetic molecules, like anti-inflammatory, antioxidative, anticollagenase, antielastase, and antimicrobial activities with lesser side effects.

## 5. Overview of Various Novel Drug Delivery Strategies

In case of dermatological pharmacotherapy, for the treatment of skin inflammatory and infectious disorders like acne, the dermal delivery of active ingredients is desirable. Topical application of antiacne agents assures many advantages over oral or intravenous administration such as it offers avoidance of first pass metabolism and elimination of gastrointestinal irritation. Skin being an effective barrier for foreign permeation encumbers the access of antiacne agents to the pathologic site, thereby decreasing its bioavailability. Therefore, the topical dosage forms against acne should be designed such that topical dosage form facilitates the delivery of active moiety to the target site that is pilosebaceous unit of the skin. With this intention, an intelligently framed delivery system encapsulating the active moiety should be developed appending specific ligands to target the active site overcoming the biological barriers.

The encapsulation of antiacne drugs in vesicular and particulate delivery system represents an innovative alternative to minimize the related side effects, while preserving their efficacy. Novel drug delivery strategies can play a pivotal role in improving the topical delivery of antiacne agents by enhancing their dermal localization with a concomitant reduction in their side effects. Research is encouraging towards a growing commitment in the development of new technologies to optimize delivery systems that may overcome technical hitches by influencing drug release, improving retention of the drug through targeting and reducing local drug toxicity, lowering dose of active agents and combination therapy, and harnessing more potent drugs which cannot clinically be utilized through conventional drug delivery. Various drug delivery carriers recently explored to beat acne vulgaris are nanoparticles, liposomes, niosomes, solid lipid nanoparticles, nanoemulsions, and nanosuspensions. In fact, their ability in improving the topical delivery of antiacne agents has been very well established by* in vitro* experiments [125, 126]. In a recent study, solid lipid nanoparticles with chitosan containing tretinoin were prepared and characterized, which showed high encapsulation efficiency, high physical stability, and absence of cytotoxicity in keratinocytes. It also exhibited antibacterial activity against* P. acnes,* thereby increasing the therapeutic efficacy of tretinoin in the topical treatment of acne [127]. Further investigations are needed, however, to allow the large scale production of novel drug delivery systems at lower costs. Finally, complementary efforts are required to validate the ability of these strategies in enhancing topical treatment of acne. A brief description of these novel carriers, their method of preparations, and advantages have been summarized in Table 2.

## 6. Conclusion

Although there are numerous drug therapies for the treatment of acne (topical, systemic retinoid, antibiotics, and keratolytics), the foremost challenge is the growing concerns of rising antibiotic resistance and dermal toxicities with existing medications. The authors praise natural remedies as an alternative against acne over these synthetic drugs. These developing natural therapies cover naturally derived drugs from active plant extracts, essential oils, and phytomolecules which are discussed in the review. However, there are certain issues allied to natural therapies, for example; while considering plant extracts, it is necessary to define the quality and safety of extracts. These problems can be resolved through standardization by advance analytical techniques (HPLC, HPTLC, GC, and LC-MS/MS). Essential oil and phytomolecule, despite having excellent bioactivity* in vitro,* demonstrate less or no* in vivo* actions due to their volatility, irritation, poor lipid solubility, or improper molecular size, resulting poor absorption, and bioavailability. These hitches could be overcome by incorporating the active moiety in some novel carrier that will reduce the chance of direct contact of active moiety with environment and skin surface; thereby, they prove to be less irritating (an advantage attributed to controlled release). Moreover, the overall cost in formulating novel carriers is lower than the conventional ones giving similar activity, because it requires less frequent administration and less intervention for toxicity. Furthermore, lack of an animal model which would purely mimic histologic and immunophenotypic characteristics of acne is another major challenge as this dreadful disease usually occurs only in humans. Depending upon the expressed cytokines in the acne lesions, an anti-inflammatory model with emphasis on major cytokines involved can be the better option. Androgens (DHEAS, testosterone, and DHT) and other related enzymes (5-*α* reductase, anticoligenase, and elastase) being the initiator of sebocyte differentiation that contributes in sebum hyperproduction as well as hyperkeratinization can be easily mimicked in small animal models as a target for novel moieties. Comedolytic and antioxidant activities in the rabbit model can be future animal targets. Also, the molecular targets that modulate several key pathological factors of acne can be aimed to optimize the therapeutic outcome. Hence, currently we can say that still a lot of expertise and experience are needed in this area as plant drugs have massive potential against* P. acnes* which should be explored through some value added drug delivery systems.

## Figures and Tables

**Table 1:** An overview of available therapies for acne and associated factors.

Class	Drug	Dose	Mode of action	Clinical evidence	Adverse effects	Reference
Retinoids	Tretinoin	0.01–0.05%	Anti comedogenic effect, indirect antimicrobiotic effect; anti-inflammatory, ability to regulate keratinocyte desquamation	Mild comedonal acne	Low grade irritant dermatitis with erythema and scaling, burning, photosensitivity	[4, 34–38, 42]
	Isotretinoin	1.0–2.0 mg/kg/day	Inhibits sebaceous gland differentiation and proliferation, reduces sebaceous gland size, suppresses sebum production, normalizes follicular epithelial desquamation, regulates keratinocyte—keratinocyte adhesion, antiinflammatory effect	Severe recalcitrant nodular acne	Cheilitis, conjunctivitis, hyper triglyceridemia, elevated serum cholesterol levels and liver enzymes, blood dyscrasias, dry eyes and mouth, photosensitivity, and pruritus; incidence/risk of teratogenicity, myalgia, arthralgia, headaches, malaise, mood swings and depression; acne fulminans, characterized by extensive erosive lesions, fever, arthralgias, leukocytosis premature epiphyseal closure in young children, asthma exacerbation, skin fragility, spectrum of ophthalmological disturbances, from dry eyes to optic neuritis	
	Adapalene	0.1%–0.3%	Anti comedogenic effect, indirect antimicrobiotic effect	Mild to moderate comedonal acne	Irritation, contraindicated in pregnant women	
	Tazarotene	0.05% or 0.1%				
	Retinol	0.04%–0.07%			Photosensitivity, xerosis, cheilitis	

Keratolytics	Azelaic acid	20%	Anticomedogenic and antimicrobial effect, reduces the production of keratohyalin granules in the pilosebaceous duct and thus normalizes the ductal hypercornification; keratolytic and anti-inflammatory	Mild to moderate acne	Only a slight sensation of burning or tingling, mild erythema	[34–36, 42]
	Benzoyl peroxide	1%–10%	Antimicrobial effect (reactive oxygen species are generated that kill bacteria by oxidizing constituents of their cell membranes), antiinflammatory, very mild anti comedogenic effects, keratolytic	Inflammatory acne, Mild to moderate acne	Irritant dermatitis with erythema and scaling, dryness, peeling, stinging, or burning, bleach hair, clothes, and bed linens	
	Salicylic acid	2%	Anti-inflammatory, topical desquamating agent, comedolytic	Comedonal acne	Peeling, hyperpigmentation, toxic inner ear damage, hypoglycemia, hypersensitivity, acute salicylate intoxication	[36, 37]
	Sulfur	1–10%	Keratolytic activity		Malodor and dry skin	
	Zinc	200 mg/day (Zn gluconate), 400 or 600 mg/day (Zn sulfate )	Bacteriostatic, inhibits chemotaxis, and may decrease tumor necrosis factor—*α* production	Mild or moderate acne, severe and inflammatory acne	Nausea, vomiting, and diarrhoea with gastrointestinal side effects	
	Alpha hydroxyl acids (Glycolic and lactic acid)	10%	Exfoliative capabilities, promotes epidermolysis, disperse basal layer melanin, increases collagen synthesis within the dermis			
	Sodium sulfacetamide	10% with 5% sulphur	Inhibiting *P. acnes *proliferation, act through competitive antagonism of para-aminobenzoic acid, halting bacterial DNA synthesis	Inflammatory lesions and comedones	Mild transient dryness, itching	

Antibiotics	Erythromycin	2%–4%	Antimicrobial effect, a significant decrease of free fatty acids of the skin surface lipids, as a marker of *P. acnes* lipase activity, indirect anticomedogenic effect, suppress leukocyte chemotaxis and bacterial lipase activity	Moderate to severe inflammatory acne	Bacterial resistance and cross resistance	[4, 34–36, 38, 42]
	Clindamycin	1%	Antibacterial, anti-inflammatory	Inflamed lesions	Antibiotic resistance, diarrhoea, pseudomembranous colitis	
	Tetracycline	500 to 1000 mg/day	Suppress leukocyte chemotaxis and bacterial lipase activity	Moderate to severe inflammatory acne	Drug-induced lupus erythematous or a dose-duration pigmentation, abdominal colic, diarrhoea and vaginal candidiasis, enamel hypoplasia and a yellowish discoloration of the forming teeth, gastrointestinal discomfort, less commonly, photosensitivity, esophagitis, pancreatitis and pseudo-porphyria	
	Minocycline	75–200 mg/day	Inhibits cytokines and matrix metalloproteinases thought to contribute to inflammation and tissue breakdown	Mild papulopustular acne	Urticaria to drug induced lupus, vertigo, dizziness, ataxia, and rarely a bluish discoloration of the skin, autoimmune hepatitis, hyper sensitivity syndrome	
	Doxycycline	50 to 100 mg twice Daily			Photosensitivity reactions, gastrointestinal disturbances	
	Trimethoprim/ Sulfamethoxazole		Anti-inflammatory effect, antibacterial, blocks dihydrofolate reductase/dihydropteroate synthetase, which ultimately diminishes bacterial purine and pyrimidine synthesis		Thrombocytopenia, agranulocytosis anemia, hypersensitivity	

Hormonal therapy	Spironolactone	50–100 mg/day	Androgen receptor blocker, which decreases androgen-stimulated sebocyte proliferation, inhibit androgen biosynthesis by decreasing type II 17*α*-hydroxysteroid dehydrogenase, therefore halting the conversion of androstenedione to testosterone, inhibition of 5 *α*-reductase and increased steroid-hormone binding globulin together resulting in 30–50% reduction in sebum excretion	Moderate acne in women	Hyperkalemia, diuretic effect, dysmenorrhea, dysphoria, breast tenderness menstrual irregularities, lethargy, headache, lightheadedness, dizziness, orthostatic hypotension, gynaecomastia in men	[35, 38, 38, 40]
	Flutamide	250–500 mg/day	Converted to its highly potent metabolite, 2-hydroflutamide, which acts to selectively inhibit the binding of dihydrotestosterone to the androgen receptor		Hepatotoxicity and GIT disturbances, hot flashes, and decreased libido	
	Cyproterone acetate	50–100 mg/day	Reduce sebum production, comedogenesis		Hepatotoxicity, breast tenderness, headache, nausea, breakthrough bleeding	
	Ethinyl estradiol	20–35 *µ*g	Reduce the production of androgens and sebum by inhibiting LH and FSH, thereby suppressing ovulation and ovarian androgen production, increasing sex hormone binding globulin and by decreasing levels of freely circulating testosterone and inhibit ovarian androgen production		Nausea, mood changes, contraindications to using OCs in an otherwise healthy woman include smoking, migraine headaches with aura and hypertension	
	Norgestimate, Norethindrone, Drospirenone	35 *μ*g	Suppress ovarian androgens and reduce bioavailable testosterone by an estrogen-mediated increase in steroid hormone binding globulin			
	prednisone	2.5 to 5 mg/d	Adrenal androgen-production blockers			

**Table 2:** Novel drug delivery systems for antiacne agent.

Novel drug delivery carrier	Drug entrapped	Method of preparation	Problem statement	Advantages	Reference
Nanoparticles	Chitosan Alginate	Ultrasonication	Low solubility	Enhanced antimicrobial activity	[128]
	Minocycline	Ion pairing	Lack of drug loading and entrapment efficiency due to hydrophilicity of the drug	Enhanced drug loading and entrapment efficiency and controlled release	[129]
	Azelaic acid	Emulsification solvent diffusion	Fewer side effects	Enhanced drug retention at PSU and stability	[130]
	Triclosan	Solvent displacement	Insufficient permeation and absorption via cutaneous route	Non-irritant to skin, enhanced stability	[131]
	Cyproterone acetate	Ultrasonication	Systemic antiandrogenic effects	Increased skin penetration and absorption	[132]
	*Garcinia mangostana *	Solvent Displacement; Ion gelation reaction	Water insolubility	Increased follicular penetration and absorption; Increased therapeutic activity	[133, 134]

Niosomes	Gallidermin	Freeze drying	For oral administration only and systemic pass demerits	Topical formulation with enhanced chemical stability	[135]
	Tretinoin	Thin film hydration	Photodegradation	Increased accumulation in superficial stratum and stability, Increased drug release and entrapment efficiencies	[136, 137]
	Rosmarinic acid	Reverse phase evaporation	Low water solubility	Increased skin retention of drug and facilitated prolong release	[121]

Liposomes	Isotretinoin	Sonication	Skin irritation, very low water solubility, difficulty to incorporate in topical base, photodegradation	Potential for skin targeting, prolonging drug release, reduction of photodegradation and skin irritation	[138]
	Clindamycin hydrochloride	Film formation	Lesser reduction in number of lesions	Enhanced antiacne activity and sustained release of drug	[139]
	Finasteride	Film formation	Only oral administration possible	Topical application with enhanced drug concentration	[140]
	Tretinoin	Film formation	Skin irritant, photo instability	Enhanced local tolerability and 5-6 times increase comedolytic activity, Reduced photo instability	[141, 142]
	Tea tree oil	Ultrasonication	Lesser absorption via follicular route	Facilitated follicular route absorption	[143]
	Salicylic acid	Thin film hydration	Skin irritant	Increased entrapment efficiency and stability	[144]
	Cyproterone acetate	—	Systemic antiandrogenic effects	Increased activity by reducing acne lesions and adverse effects	[145]
	Benzoyl peroxide	Film hydration	Skin irritation	Improved antibacterial activity, reduced irritation	[146]

Solid lipid nanoparticles	Isotretinoin	Microemulsification	Teratogenicity, mucocutaneous problems like cheilitis, dermatitis, conjunctivitis, blepharitis, skin fragility and xerosis, psychological disorders, erythema, dryness, itching, stinging, skin peeling	Reduced dermal irritation, increased therapeutic performance	[147]
	Retinoic acid	Hot melt homogenization	Sensitive to sunlight, eczematous irritation, erythema, interaction with other applied products	Comedolytic effect, reduction in RA induced irritation	[148]
	Neem oil	Double emulsification	Lesser drug absorption	Prolonged treatment of acne	[149]
	Tretinoin	Hot high pressure homogenization	Skin irritation and chemical instability	High encapsulation efficacy, physical stability and absence of cytotoxicity	[127]
	Terbinafine hydrochloride	Solvent Injection	Longer duration of treatment	Controlled release, drug targeting	[150]

Nanosuspension	Tretinoin	Precipitation	Poor water solubility and photostability	Improved drug permeation and UV irradiation stability	[151]

Nanoemulsion	Tretinoin, Tetracycline	Sonication	Skin irritation, a burning sensation, and peeling	Enhanced drug permeation and antibacterial activity	[152]

Nano lipid carriers	Tretinoin, Tetracycline	Sonication	Skin irritation, a burning sensation, and peeling	Enhanced drug permeation and antibacterial activity	[152]

Microemulsions	Azelaic acid	—	Large and frequent dosing	Enhanced stability	[153]
	Niflumic acid	Homogenization	Weak solubility in oil and aqueous phases	Increased bioavailability at lesser concentration	[154]
	Retinoic acid	Homogenization	Systemic side effects	Enhanced skin accumulation of retinoic acid	[155]

Microspheres	Benzoyl peroxide	Emulsification	Skin irritation	Appropriate reduction in *P*. *acnes * count, reduced skin irritation	[156]
	Retinoid	Emulsification	Skin irritation and instability	Reduced irritation and enhanced stability	[157]

Hydrogel Patches	Triclosan	Film gelation	Insufficient permeation and absorption via cutaneous route	Enhanced transdermal penetration	[158]

## Abbreviations

NCEs: New chemical entities
MIC: Minimum inhibitory concentration
MBC: Minimum bactericidal concentration
DHEAS: Dehydroepiandrosterone
DHT: Dihydrotestosterone
TLR2: Toll like receptors
IL: Interleukin
GMCSF: Granulocyte macrophage colony stimulating factor
CYP21: Cytochrome P21
PPAR*γ*: Peroxisome proliferator activating receptor
MMP: Matrix metalloproteinase
IGF: Insulin growth factor
FGFR: Fibroblast growth factor receptor
DP IV: Dipeptidyl peptidase IV
APN: Aminopeptidase N
CRH: Corticotrophin releasing hormone
HPLC: High performance liquid chromatography
ELISA: Enzyme linked immunosorbent assay
NOS: Nitric oxide synthase
COX: Cyclooxygenase
PGE2: Prostaglandin E2
LPS: Lipopolysaccharide
NDDS: Novel drug delivery system.
